# Analysis of the Efficacy and Prognosis of Microsurgery and Transarterial Embolization in the Treatment of High-Grade Dural Arteriovenous Fistulas

**DOI:** 10.1155/emmi/4968557

**Published:** 2025-11-24

**Authors:** Cheng Qiu, Yijia Zhang, Lijiu Chen, Yonghui Xu, Tianci Huang, Guangxu Zhang, Zhiqiang Yu, Jinbing Zhao, Shengxue He

**Affiliations:** ^1^Nanjing Comprehensive Stroke Center, Affiliated Nanjing Brain Hospital of Nanjing Medical University, Nanjing, China; ^2^School of Medicine, Hangzhou City University, Hangzhou, China

**Keywords:** Cognard types, fistula occlusion, high-grade dural arteriovenous fistulas, microsurgical treatment, transarterial endovascular embolization

## Abstract

**Objective:**

To evaluate the surgical outcomes of microsurgical treatment and transarterial endovascular embolization for dural arteriovenous fistulas (DAVFs).

**Methods:**

A retrospective analysis was conducted on 47 patients diagnosed with high-grade DAVFs (Cognard types 2b, 2a + b, 3, 4, and 5) between June 2019 and June 2022 at Nanjing Brain Hospital, affiliated with Nanjing Medical University. These patients underwent either microsurgery or endovascular embolization. Postoperative efficacy, surgical complications, and related prognostic factors were compared between the two groups. The primary endpoint was a postoperative modified Rankin Scale (mRS) score > 3. Secondary outcomes included angiographic confirmation of fistula occlusion, complication rates, and neurological deficits.

**Results:**

In the microsurgical treatment group, complete fistula occlusion was achieved in 23 patients (85.185%), while 12 patients (60.000%) in the transarterial embolization group (single-session treatment) achieved complete occlusion. Although microsurgical treatment demonstrated a higher occlusion rate, this difference did not reach statistical significance (*p*=0.051). The median follow-up duration for all patients was 12 months (IQR 1–38 months). During follow-up, 2 patients (4.255%) with residual untreated fistulas developed severe neurological deficits. In contrast, none of the patients with single fistulas developed severe postoperative complications.

**Conclusion:**

Microsurgical treatment demonstrated a higher rate of complete fistula occlusion compared to single-session transarterial embolization in patients with DAVFs, although this difference was not statistically significant. Both treatment modalities showed acceptable safety profiles. Patients with single fistulas showed a lower risk of severe postoperative neurological deficits compared to those with multiple fistulas. This study provides important insights into the emergency treatment of DAVF.

**Trial Registration:**

Chinese Registry of Clinical Trials: ChiCTR2300072890

## 1. Introduction

Dural arteriovenous fistulas (DAVFs) are abnormal arteriovenous shunts between dural arteries and dural sinuses, meningeal veins, or cortical veins [1]. Intracranial DAVFs are rare and can occur at any location where the dura mater is present. The estimated incidence is 0.51 cases per 100,000 person-years [2]. Fistulas with cortical venous drainage, classified as high-grade DAVFs [3, 4], carry an annual risk of intracranial hemorrhage (ICH) ranging from 1.5% to 13.0% [5–8]. Treatment strategies for these lesions include fistula occlusion via endovascular therapy (EVT), stereotactic radiosurgery (RT), microsurgery, or multimodal approaches. Reported occlusion rates vary from 60.0% to 95.0% [9–14], reflecting differences in patient characteristics, follow-up duration, treatment modality, technical approach, and attrition rates. Despite extensive research, outcomes regarding postoperative rebleeding and long-term prognosis after DAVF treatment remain inconclusive. Considering the risk of acute ICH and neurological deterioration, high-grade DAVFs warrant emergency evaluation and time-sensitive decision-making in the emergency.

Follow-up data from our center aims to address this gap and contribute to the existing evidence. At our institution, the primary treatment modalities for DAVFs are microsurgical resection and endovascular embolization. This study retrospectively analyzes the clinical data of 47 patients diagnosed with high-grade DAVFs at Nanjing Brain Hospital, affiliated with Nanjing Medical University, between June 2019 and June 2022. Our objective was to evaluate the efficacy of these treatments and provide evidence-based insights to optimize clinical management.

## 2. Materials and Methods

This study included a total of 47 patients diagnosed with DAVFs. Among them, 27 patients underwent microsurgical resection, while 20 patients received endovascular embolization. The cohort consisted of 37 males and 10 females, with ages ranging from 22 to 76 years (mean age: 47.070 ± 11.320 years).

### 2.1. Inclusion Criteria

A confirmed diagnosis of high-grade DAVFs (Cognard types 2b, 2a + b, 3, 4, and 5 [3]) based on cerebral digital subtraction angiography (DSA). (2) All patients and their families participated in preoperative counseling, selected the treatment modality (microsurgery or embolization) according to their preferences and clinical condition, and provided written informed consent.

### 2.2. Exclusion Criteria

(1) Low-grade DAVFs (Cognard type I). (2) Patients lost to follow-up after treatment. (3) Age < 18 years.

### 2.3. Endovascular Embolization

In our series, all endovascular procedures were performed via a transarterial approach. Embolic material and technique selection were individualized according to arterial anatomy and the presence of dangerous anastomoses. Overall, Onyx (Onyx-18 or Onyx-34) was used in 14 of 20 endovascular procedures (70.000%). NBCA (n-butyl cyanoacrylate) was used in 3 procedures (15.000%), and coils were used as adjunctive anchors in 5 procedures (25.000%). Balloon-assisted occlusion was employed in 2 procedures (10.000%) to protect adjacent important arteries and reduce reflux risk. The pressure-cooker technique was not used in our series.

### 2.4. Imaging Evaluation

All patients received a comprehensive neuroimaging assessment. We employed DSA as the gold standard for DAVF diagnosis and Cognard classification determination. (1) Preoperative DSA: On admission, DSA was performed in all patients to confirm DAVF diagnosis and establish classification according to the Cognard grading system. (2) Postoperative DSA follow-up: We conducted repeat DSA examinations during hospitalization follow-up to assess treatment outcomes, particularly verifying complete fistula occlusion and confirming absence of early venous drainage.

### 2.5. Primary and Secondary Outcomes

We defined the primary outcome as postoperative modified Rankin Scale (mRS) scores exceeding 3 during follow-up, with patient monitoring extending through September 2024. Secondary outcomes encompassed angiographic fistula occlusion, treatment-associated complications, and newly developed neurological deficits. We classified neurological symptoms based on duration: symptoms resolving within 6 months post-treatment were designated as temporary neurological deficits, while those persisting beyond 6 months were classified as permanent neurological deficits. Single seizure episodes were excluded from new neurological deficit categorization.

### 2.6. Treatment Strategies

The surgical approach for each patient was determined based on the anatomical location of the DAVF, the characteristics of the feeding arteries, and the patterns of venous drainage. Of the 47 patients, 27 underwent microsurgery, 20 received endovascular embolization, and 3 patients underwent both procedures.

#### 2.6.1. Microsurgical Treatment (Figure 1)

General principle: the fistula orifice should be addressed first, followed by coagulation of the feeding arteries to prevent recurrence. The strategy begins by focusing on the fistula orifice after fully exposing the draining vein, the dural transition zone, and the fistula orifice area. A preliminary assessment is made based on venous coloration: arterialized veins appear red, turgid, and well-distended, with pulsatile blood flow sometimes visible within the lumen. Next, indocyanine green (ICG) angiography is performed: 25 mg of ICG is dissolved in 5 mL of sterile water for injection and rapidly administered via the femoral vein. Fluorescence microscopy is used to observe early-filling veins and/or refluxing veins. The timing and direction of venous contrast enhancement are used to identify abnormal veins. Following this, temporary occlusion of the fistula orifice is performed near the dura. Repeat ICG angiography is performed, showing an absence of contrast in the previously early-filling veins, confirming complete occlusion of the fistula. The feeding arteries are divided as completely as possible, followed by electrocoagulation of the affected dura mater and dural venous sinuses. Arterialized draining veins are severed to prevent recurrence. The feeding arteries are addressed systematically from superficial to deep layers, starting with the superficial arteries (e.g., the occipital artery) to avoid arterial rupture and hemorrhage during bone flap elevation. For complex DAVFs with multiple fistula orifices and multifocal arterial supply, a sectional craniotomy may be performed during bone flap elevation to facilitate control of dural bleeding. The feeding arteries often branch into small, tortuous arterioles before converging into the fistula at the superior sagittal sinus, transverse sinus, or sigmoid sinus, sometimes forming sheets or even encircling the sinus wall. To disconnect these feeders, coagulation of the venous sinus is typically required. While coagulating the transverse sinus dura is relatively straightforward, DAVFs involving the sigmoid sinus pose greater challenges. Their feeders often originate from the external carotid artery (e.g., ascending pharyngeal artery, occipital artery branches at the jugular foramen), necessitating a combined presigmoid approach. Even with this approach, achieving complete coagulation may be difficult. Maximal sinus coagulation should be prioritized to minimize recurrence risk. The detailed procedure is clearly demonstrated in the accompanying surgical video (Supporting File 1).

#### 2.6.2. Principle of Endovascular Embolization

**Figure 2:**
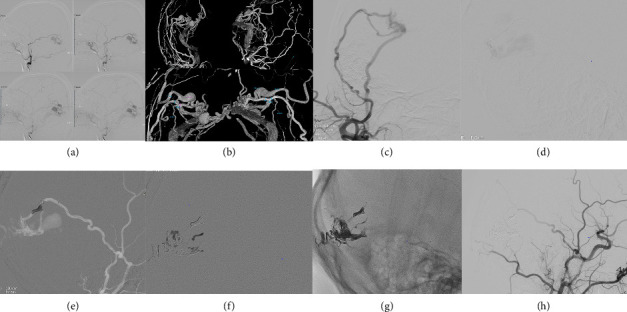
A 46-year-old female patient was admitted to the hospital with complaints of tinnitus. Digital subtraction angiography (DSA) and 3D-DSA revealed a dural arteriovenous fistula supplied by the posterior branch of the middle meningeal artery, draining into the cortical vein and transverse-sigmoid sinus system (a, b). Intraoperative magnifying angiography was performed to further delineate the vascular architecture (c). Microcatheter angiography confirmed that the catheter tip successfully reached the fistula site (d). Subsequently, a spring coil was deployed at the proximal end of the fistula using another microcatheter to serve as an anchor for subsequent embolization (e). Bio-glue was satisfactorily distributed within the feeding arteries, the fistula itself, and the draining veins (e, f). Postoperative DSA demonstrated the absence of the fistula and the abnormal draining vein (g, h).

**Figure 3:**
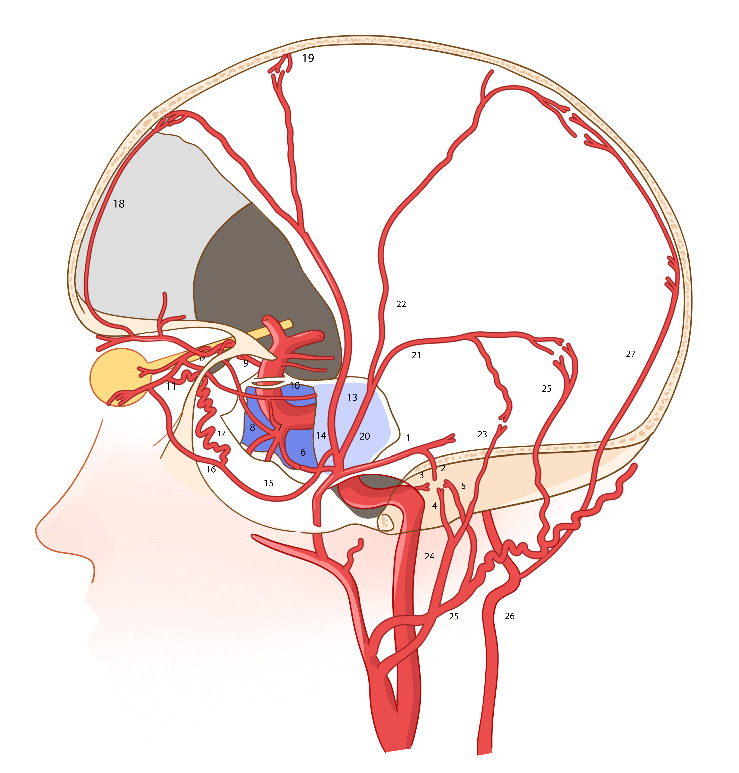
(1) MMA petrosal branch; (2) superior tympanic artery; (3) caroticotympanic artery; (4) inferior tympanic artery; (5) posterior tympanic artery; (6) MMA cavernous sinus branch; (7) internal carotid cavernous sinus inferior lateral trunk; (8) deep recurrent meningeal artery; (9) ophthalmic artery; (10) tentorial artery; (11) lacrimal artery; (12) superficial recurrent meningeal artery; (13) meningohypophyseal trunk; (14) frontal branch MMA; (15) medial branch of frontal branch MMA; (16) lateral meningolacrimal artery; (17) medial sphenoid sinus artery; (18) anterior falx artery; (19) the opposite side MMA branch.

The principle of endovascular embolization is to advance the catheter to the fistula orifice and occlude both the fistula and the draining vein (Figure 2). Comprehensive cerebral angiography and microcatheter superselective angiography are essential to identify the DAVF's feeding arteries, fistula orifice location, venous drainage patterns, and detect dangerous anastomoses that could lead to nontarget embolization. The success of embolization depends on the effective diffusion of embolic agents. The middle meningeal artery (MMA), with its interconnected branches, plays a key role in the spread of liquid embolic agents (e.g., Onyx) and is considered the gold standard route for transarterial endovascular treatment of DAVFs (Figure 3).

At the origin of the MMA bifurcation, a cavernous sinus branch arises anteriorly and a petrosal branch posteriorly. The petrosal branch gives rise to the superior tympanic artery, which enters the middle ear and anastomoses with the carotid artery branch originating from the petrous segment of the internal carotid artery. This anatomical configuration corresponds to the inferior tympanic artery from the ascending pharyngeal artery and the posterior tympanic artery from the occipital artery. The inferior lateral trunk of the cavernous sinus may anastomose with the contralateral cavernous sinus branch, in opposition to the petrosal branch. Additionally, this trunk can connect to the ophthalmic artery through the deep meningeal recurrent branch of the ophthalmic artery. The inferior lateral trunk of the cavernous sinus artery, MMA, and ophthalmic artery may also interconnect via the tentorial artery.

The marginal tentorial artery typically originates from the meningohypophyseal trunk, although variations may cause it to arise from the superficial recurrent meningeal branch of the ophthalmic artery (via the lacrimal artery). At the pterional region, the MMA bifurcates into anterior and posterior branches, with the medial branch potentially giving rise to the lateral meningolacrimal artery and the medial sphenoid sinus artery, both of which may anastomose with the lacrimal artery. Clinicians should be aware of the potential dangerous anastomoses between the MMA and either the ophthalmic artery or the inferior lateral trunk of the cavernous sinus.

The frontal branch of the MMA extends to the convexity and follows the coronal suture toward the midline, where it anastomoses with the anterior falciparum artery (a branch of the anterior ethmoidal artery from the ophthalmic artery) and with the contralateral MMA across the midline. The posterior branch of the MMA is further subdivided into the parieto-occipital branch and the petrosquamosal branch. The petrosquamosal branch anastomoses with the jugular bulbar branch of the ascending pharyngeal artery and the mastoid branch of the occipital artery. Additionally, the parieto-occipital branch overlaps with the posterior meningeal artery from the vertebral artery in the critical zone.

### 2.7. Statistical Methods

Data were analyzed using SPSS version 26.0. Continuous variables following a normal distribution are presented as mean ± standard deviation ($\bar{x}\pm s$), with intergroup comparisons conducted using the *t*-test. Categorical variables are expressed as frequencies and percentages, and intergroup comparisons were performed using the chi-square test. Significant variables between the two groups were further analyzed using multivariate logistic regression. A *p* value of less than 0.050 was considered statistically significant.

## 3. Results

### 3.1. Analysis of Clinical Data

A total of 50 patients met the inclusion criteria, but 3 patients were not treated due to poor general condition or unsuitable individual factors. As a result, the final study population consisted of 47 patients. Of these, 21.277% were female, and the mean age was 47.070 ± 11.320 years. High-grade DAVFs are associated with a high risk of bleeding, and 13 patients (27.660%) presented with intracerebral hemorrhage as the initial symptom. The most common sites of DAVF were the transverse/sigmoid sinus, tentorial/petrosal sinus, and superior sagittal sinus, which together accounted for 89.000% of all cases. The distribution of DAVF types was as follows: Cognard IIb in 11 cases (23.404%), Cognard IIa + b in 9 cases (19.149%), Cognard III in 12 cases (25.532%), Cognard IV in 10 cases (21.277%), and Cognard V in 5 cases (10.638%). Complete patient baseline characteristics are presented in Table 1.

### 3.2. Analysis of Surgical Efficacy

A total of 47 patients underwent surgical treatment, including 27 (57.447%) who received microsurgical treatment and 20 (42.553%) who underwent endovascular treatment. All procedures were performed via an arterial approach, without staged embolization. Among these, 3 patients underwent two operations, and 1 patient underwent two microsurgical procedures. After a median follow-up of 16 months, complete fistula occlusion was achieved in all cases. One patient underwent both endovascular and microsurgical treatments, achieving complete occlusion after 11 months. Another patient underwent two endovascular procedures, but the fistula remained present after 3 months of follow-up.

Among the patients who received endovascular treatment, all procedures employed a purely transarterial approach, with the MMA being the primary route for embolization when involved in fistula supply. Baseline characteristics differed between groups. The microsurgical treatment group had fewer males (88.889% vs. 65.000%, *p*=0.048), varied Cognard distributions (*p*=0.047), higher single spout prevalence (70.370% vs. 25.000%, *p*=0.002), and better initial occlusion rates (85.185% vs. 60.000%, *p*=0.051). Embolization was preferred for multispout fistulas or those with complex venous patterns, while microsurgical treatment suited single-spout lesions with direct access.

In the microsurgical treatment group, complete fistula occlusion was achieved in 23 patients (85.185%), while 12 patients (60.000%) in the transarterial embolization group (single-session treatment) achieved complete occlusion. The difference between the two groups did not reach statistical significance (*p*=0.051; Table 2). Postoperative outcomes with significant neurological deficits (mRS ≥ 3) occurred in 4 patients, with mRS scores of 5, 4, 4, and 3. Among these, 3 patients (11.111%) had undergone microsurgical treatment, and 1 patient (5.000%) had received endovascular treatment. The difference between the two groups was statistically significant (*p*=0.009; Table 2).

After excluding patients with preoperative intracerebral hemorrhage and those with baseline mRS scores > 3, only 1 case (3.704%) in the microsurgical treatment group experienced significant neurological deficits (mRS ≥ 3), while the remaining 26 patients (96.296%) had favorable surgical outcomes. The choice of surgical modality (microsurgical treatment vs. endovascular treatment) showed no statistically significant association with the presence of MMA involvement or preoperative hemorrhage (*p* > 0.050).

### 3.3. Hemorrhage and Prognostic Risk

After definitive treatment, complete follow-up data were available for all 47 treated patients, with a median follow-up duration of 12 months (interquartile range [IQR]: 1–38 months). Only 5 patients (10.638%) had follow-up durations less than 6 months; in these cases, unresolved symptoms at the last assessment were classified as permanent deficits to align with outcome definitions. During follow-up, 2 patients (4.255%) with multiple untreated residual fistulas experienced poor outcomes accompanied by severe neurological deficits.

The two patients with poor prognosis (mRS > 3) had multiple untreated residual fistulas: one (Cognard IV, Borden 3) experienced rebleeding postmicrosurgery leading to permanent quadriparesis; the other (Cognard IIa + b, Borden 2) developed venous infarction after partial embolization, resulting in cognitive decline [15]. In contrast, none of the patients with single fistulas developed severe postoperative complications.

**Figure 4:**
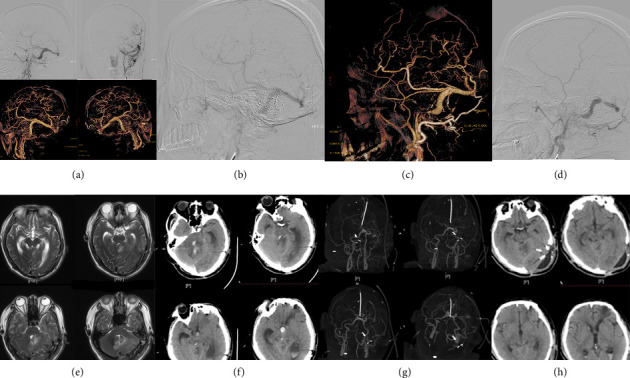
The patient, a 57-year-old male, was admitted to the hospital with a chief complaint of “headache persisting for more than three months.” (a) Preoperative cerebral angiography revealed the presence of a dural arteriovenous fistula involving the left transverse and sigmoid sinuses. (b, c) Postembolization angiographic imaging demonstrated partial obliteration of the dural arteriovenous fistula following the initial endovascular intervention. (d) Subsequent angiography after the second craniotomy, performed for definitive resection of the fistula, confirmed complete elimination of cortical retrograde venous drainage. (e) One month postsurgery, the patient developed acute severe headache. A follow-up cranial MRI revealed cerebral edema in the left cerebellar hemisphere accompanied by a small intraparenchymal hemorrhage. (f, g) The patient subsequently underwent a surgical procedure comprising right external ventricular drainage, intracranial hematoma evacuation, and cerebellar decompressive craniectomy; during this operation, the transverse sinus was ligated. (h) A cranial CT scan performed 6 months after the final surgical intervention showed no evidence of hydrocephalus, and the patient exhibited favorable neurological recovery.

**Figure 5:**
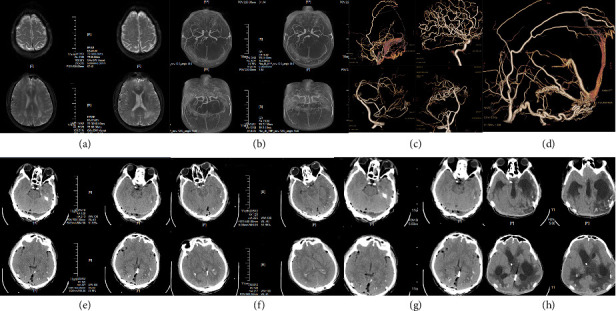
The patient, a 54-year-old male, was admitted to the hospital with a chief complaint of “gradually worsening low mood over the past year, with significant exacerbation in the last two months.” (a, b) Brain imaging revealed multiple abnormal vascular structures, including arteriovenous fistulas, accompanied by white matter edema, congestion, and demyelinating changes. (c, d) Cerebral angiography confirmed the presence of dural arteriovenous fistulas involving the bilateral transverse sinuses, sigmoid sinuses, and the torcular herophili (confluence of sinuses). (e) On the first postoperative day, the patient remained in a drowsy state of consciousness; a follow-up cranial CT scan showed no evidence of intracranial hemorrhage or cerebral infarction. (f) On the first postoperative night, the patient experienced a sudden loss of consciousness, and an emergency cranial CT scan revealed diffuse cerebral edema, more pronounced than preoperatively. (g) The patient subsequently underwent bilateral frontotemporal, parietal, and cerebellar decompressive craniectomy, and postoperative cranial imaging demonstrated improvement in cerebral edema compared to preintervention scans. (h) Despite these interventions, the patient's overall prognosis remained unfavorable.

Following endovascular embolization, incomplete occlusion of the fistula occurred. Hemorrhage developed 1 month after treatment, necessitating subsequent surgical intervention. At 6 month follow-up, the patient's functional outcome was assessed as an mRS score of 3, indicating moderate disability and requiring assistance with daily activities (Figure 4). Another patient (4.255%) developed postoperative cerebral edema secondary to recurrent infections. This patient initially presented with nonhemorrhagic neurological deficits and had a Cognard IIa + b DAVF with multiple fistula orifices. Following microsurgical treatment, incomplete fistula occlusion occurred, leading to postoperative recurrent infections and progressive cerebral edema. Despite multiple salvage surgeries, the patient's condition deteriorated, and at 12 month follow-up, the functional outcome was assessed as an mRS score of 5, indicating severe disability requiring constant nursing care (Figure 5).

After excluding the two cases with multifocal fistula orifices (a known risk factor for complications), patients with single-orifice fistulas showed no significant risk of severe postoperative complications. Additionally, a patient with a complex DAVF involving multiple fistula orifices in the left frontotemporal region developed postoperative seizures, which were successfully controlled with antiepileptic medication. During endovascular embolization with Onyx, the patient experienced sudden bradycardia and hypotension, this was highly suggestive of the trigeminocardiac reflex (TCR). Immediate atropine administration stabilized the hemodynamic parameters.

## 4. Discussion

The etiology of DAVFs remains debated, though most studies support the hypothesis that DAVFs are acquired lesions rather than congenital [16]. Emerging evidence links their development to pathological processes such as trauma, prior surgery, dural sinus thrombosis, and hypercoagulable states. The management of low-grade DAVFs (Cognard I-II) remains contentious. Multiple retrospective studies report an annual risk of severe complications (e.g., ICH) as low as 0.000%–0.600% [17] in untreated or partially embolized cases, raising questions about the necessity of aggressive intervention for asymptomatic or minimally symptomatic patients. However, Shen SC et al. [18] reported 20 cases of high-grade DAVF with ICH as the first symptom, with a rebleeding rate as high as 35.000% during an average waiting period for surgery of 20 days. Our center's results, showing that 13 patients (27.660%) presented with intracerebral hemorrhage as the initial symptom, were similar. High-grade DAVFs carry a significantly higher risk of bleeding and require active surgical intervention. From an emergency-care perspective, these data support managing high-grade DAVFs as neurovascular emergencies by prioritizing early ED triage, urgent angiographic diagnosis, and expedited definitive occlusion to reduce early rebleeding and neurological deterioration.

The selection of treatment for DAVF is primarily determined by the severity of symptoms, neurological manifestations, and most crucially, the pattern and type of venous drainage. The ultimate goal of treatment is to achieve complete occlusion of the fistula. A key challenge lies in the accurate diagnosis and localization of the fistula [19]. Misidentification of the fistula, which could obstruct normal venous outflow, may lead to catastrophic outcomes, including cerebral hemorrhage. Treatment options include conservative management (e.g., compression therapy for the carotid or occipital arteries), microsurgical intervention, endovascular embolization (via transarterial and transvenous approaches), and radiation therapy [20]. Complex DAVFs may require a combination of these modalities.

In our center, 47 patients with high-grade DAVFs underwent surgical treatment. Among those treated microsurgically, 23 (85.185%) achieved complete fistula obliteration, while 12 (60.000%) achieved complete fistula embolization through arterial endovascular treatment as a single modality. Halbach et al. reported a cure rate of 59.000% for transarterial embolization. Although transarterial embolization may not always achieve complete occlusion, it effectively reduces the blood supply to DAVFs and alleviates clinical symptoms. While microsurgical treatment demonstrated a higher occlusion rate compared to single transarterial endovascular treatment, this difference did not reach statistical significance (*p*=0.051). This finding suggests that both treatment modalities can be effective, with the choice depending on individual patient factors, anatomical considerations, and institutional expertise.

A recent systematic review and meta-analysis comparing endovascular versus surgical treatment for spinal DAVF highlights that comparative outcome studies can be influenced strongly by fistula anatomy, treatment selection bias, and center technique expertise [21]. Although spinal and cranial DAVFs differ in anatomy and treatment routes, the study's methodological lessons are applicable: balanced baseline comparison, stratification by fistula complexity, and reporting of technique specifics are essential to interpret comparative efficacy. It emphasizes these methodological points and highlights the need for multicenter, technique-stratified studies in cranial DAVFs.

In microsurgical treatment, the presence of a DAVF can often be assessed based on the surgeon's experience. Arterialized veins, which are typically red and fully distended, serve as key indicators. Additionally, the development or retrograde flow of these veins can be observed in advance under a fluorescence microscope using ICG. This enables the surgeon to occlude the fistula, with repeat ICG angiography confirming complete occlusion. Endovascular treatment of DAVFs can be performed using either an arterial or venous approach. The venous approach has shown promise in improving embolization rates; however, it carries the risk of bleeding due to cortical venous return disorders following venous sinus occlusion. In contrast, the arterial approach, often via the MMA, is generally considered the optimal route. The MMA's extensive communication branches facilitate the diffusion of embolic agents, such as biological glue, enhancing treatment efficacy. However, numerous dangerous anastomoses require meticulous control of glue injection to prevent complications, which can affect the overall occlusion rate of the fistula.

The relatively lower occlusion rate of transarterial endovascular embolization may be attributed to complex anatomy and high-risk anastomoses. To minimize complications, it is crucial to protect key arteries during embolization. Notable high-risk anastomoses are as follows: (1) The cavernous branch of the MMA, which may connect to the ophthalmic artery through the deep meningeal recurrent branch of the ophthalmic artery. (2) The inferior lateral trunk of the cavernous sinus, which can communicate with the ophthalmic artery via the marginal tentorial artery and the superficial meningeal recurrent branch of the ophthalmic artery. (3) The medial branch of the MMA, which branches into the lateral meningolacrimal artery and medial sphenoid sinus artery, both of which can connect to the lacrimal artery. (4) The frontal branch of the MMA, which anastomoses with the anterior ethmoidal artery of the ophthalmic artery via the anterior falciparum artery. By avoiding these high-risk anastomoses, surgical risks can be reduced, though this may lead to a lower fistula occlusion rate.

During the median follow-up period of 12 months (IQR 1–38 months), two cases of severe complications were observed. Both involved complex DAVFs with multiple fistulous connections. Postoperative imaging revealed incomplete occlusion of the fistulas, with only partial treatment achieved. The arterial pressure remained unaltered, which could increase the hemodynamic burden on the untreated fistulas, potentially leading to rebleeding or impaired venous outflow. In cases of multiple fistulas, it is essential to address all fistulous connections as comprehensively as possible or to effectively reduce the arterial inflow to minimize the risk of complications.

This study does have some limitations. First, being a retrospective study, it inherently has design limitations when compared to prospective studies. Second, all patients were recruited from a single institution, Nanjing Brain Hospital, which may limit the generalizability of the findings. Multicenter collaboration would enhance the reliability of the results. Third, the sample size in this study was relatively small, which may have limited the statistical power to detect differences between treatment groups. Future studies should expand the sample to allow for more comprehensive analyses and potentially reveal significant differences in treatment outcomes.

## 5. Conclusion

DAVFs are relatively rare intracranial vascular malformations, characterized by distinctive pathophysiological mechanisms, clinical manifestations, and imaging findings. High-grade DAVFs are particularly concerning due to their high risk of cerebral hemorrhage, requiring prompt and aggressive surgical intervention. Based on our single-center experience, microsurgical treatment demonstrated a higher occlusion rate compared to single-session transarterial endovascular embolization (85.185% vs. 60.000%), although this difference did not reach statistical significance (*p*=0.051). Both treatment modalities showed acceptable safety profiles, with the choice of approach depending on individual patient factors, fistula complexity, and institutional expertise. Patients with single-orifice DAVFs showed favorable outcomes regardless of treatment modality, while those with multiple fistula orifices carried higher risks of complications and incomplete treatment. This study provides important insights into the emergency treatment of DAVF.

## Figures and Tables

**Figure 1:**
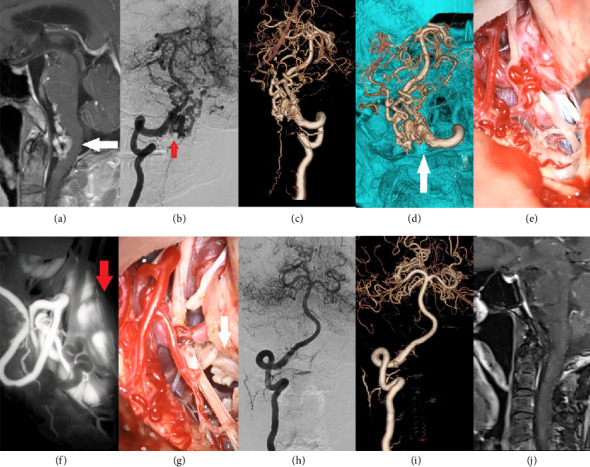
A 69-year-old male patient was admitted to the hospital due to complaints of dizziness. Cranial magnetic resonance imaging revealed an abnormal vascular shadow in the foramen magnum region (a). Digital subtraction angiography (DSA) demonstrated that the meningeal branch of the vertebral artery provided arterial supply, while the large draining vein was located in the medulla oblongata, posterior to the upper cervical spinal cord, and anterior and posterior veins of the spinal cord were also involved (b, c). 3D-DSA analysis confirmed that the fistula was situated at the left margin of the foramen magnum and was supplied by the meningeal branch of the vertebral artery; therefore, a far lateral surgical approach was selected (d). The anatomical structure of the dural arteriovenous fistula was successfully exposed. Intraoperative indocyanine green (ICG) fluorescence angiography indicated the presence of multiple advanced draining veins (e, f), which were subsequently occluded, and the reflux veins were transected (g). Postoperative DSA and cranial magnetic resonance imaging confirmed complete resolution of the fistula with no residual abnormal vascular shadows (h, i, j).

**Table 1:** Baseline data.

Parameter	All patients (*N* = 47) *n* (%)
Age (years), mean ± SD	22–76, 47.070 ± 11.320
Female	10 (21.277)
Male	37 (78.723)
Hemorrhage at initial presentation	13 (27.660)
Cognard classification^†^	
II b	11 (23.404)
II a + b	9 (19.149)
III	12 (25.532)
IV	10 (21.277)
V	5 (10.638)
Fistula occlusion	35 (74.468)
Fistula location	
Anterior cranial base	5 (10.638)
Middle cranial base	17 (36.170)
Posterior cranial base	25 (53.191)

^†^Cognard classification determined by DSA.

**Table 2:** Comparison of microsurgical and endovascular treatment characteristics.

Parameter	All treatments (*N* = 47) *n* (%)	Microsurgical treatment (*N* = 27) *n* (%)	Endovascular treatments (*N* = 20) *n* (%)	*p* value
MMA.blood.supply	29 (61.702)	16 (59.259)	13 (65.000)	0.689
Single.spout	24 (51.064)	19 (70.370)	5 (25.000)	**0.002**
Preoperative.hemorrhage	13 (27.660)	10 (37.037)	3 (15.000)	0.095
Fistula occlusion	35 (74.468)	23 (85.185)	12 (60.000)	0.051
Cognard.type				
II b	11 (23.404)	3 (11.111)	8 (40.000)	0.047
IIa + b	9 (19.149)	7 (25.926)	2 (10.000)	
III	12 (25.532)	6 (22.222)	6 (30.000)	
IV	10 (21.277)	6 (22.222)	4 (20.000)	
V	5 (10.638)	5 (18.519)	0 (0.000)	
Post-mRS ≥ 3	4 (8.511)	3 (11.111)	1 (5.000)	**0.009**

*Note:* The bold values indicate *p* values below 0.05.

## Data Availability

All data included in this study are available by contacting with the corresponding authors.

## Supporting Information

Supporting File 1. Video records of Microsurgical Treatment.
